# Thrombophilic Genetic Factors PAI-1, MTHFRC677T, V Leiden 506Q, and Prothrombin 20210A in Noncirrhotic Portal Vein Thrombosis and Budd-Chiari Syndrome in a Caucasian Population

**DOI:** 10.1155/2013/717480

**Published:** 2013-12-18

**Authors:** Mario D'Amico, Pietro Sammarco, Linda Pasta

**Affiliations:** ^1^Department of Biopathology and Medical and Forensic Biotechnologies and Biomedical Department of Internal and Specialist Medicine, University of Palermo, Palermo, Italy; ^2^Department of Oncology and Hematology, Cervello Hospital, Via Trabucco 180, 90146 Palermo, Italy; ^3^Department of Medicine, Cervello Hospital, Via Trabucco 180, 90146 Palermo, Italy

## Abstract

Thrombophilic genetic factors PAI-1, MTHFRC677T, V Leiden 506Q, and Prothrombin 20210A were studied as risk factors in 235 Caucasian subjects: 85 patients with abdominal thrombosis (54 with portal vein thrombosis (PVT) and 31 with Budd-Chiari syndrome (BCS) without liver cirrhosis or hepatocellular carcinoma) and 150 blood bank donors. Seventy-five patients with PVT/BCS showed associated disease or particular clinical status (46 PVT/29 BCS): 37 myeloproliferative neoplasm (20 PVT/17 BCS), 12 abdominal surgery (10 PVT/2 BCS), 10 contraception or pregnancy (6 PVT/4 BCS), 7 abdominal acute disease (6 PVT/1 BCS), and 9 chronic disease (4 PVT/5 BCS); ten patients did not present any association (8 PVT/2 BCS). PAI-14G-4G, MTHFR677TT, and V Leiden 506Q were significantly frequent (OR 95% CI and *χ* ^2^ test with *P* value) in abdominal thrombosis; in these patients PAI-14G-4G and MTHFR677TT distributions deviated from that expected from a population in the Hardy-Weinberg equilibrium (PAI-1: *χ* ^2^ = 13.8, *P* < 0.001; MTHFR677: *χ* ^2^ = 7.1, *P* < 0.01), whereas the equilibrium was respected in healthy controls. V Leiden Q506 and Prothrombin 20210A were in the Hardy-Weinberg equilibrium both in patients with abdominal thrombosis and healthy controls. Our study shows an important role of PAI-14G-4G and MTHFR677TT in abdominal thrombosis without liver cirrhosis or hepatocellular carcinoma.

## 1. Introduction 

Thrombophilic genetic factors (THRGFs) such as PAI-1, MTHFR677, V Leiden 506Q, and Prothrombin 20210A mutations have been studied in patients with abdominal thrombosis, but never in the same study.

We have recently published two studies on the prevalence of these THRGFs in liver cirrhosis and hepatocellular carcinoma: MTHFR677TT was highlighted as a significant risk factor for PVT in liver cirrhosis [1], but PAI-1 was not analyzed in the first study; in the second study [2] MTHFR677TT, PAI-1 4G-4G, and Prothrombin 20210A were found to be significant risk factors in hepatocellular carcinoma, mainly in the presence of portal vein thrombosis (PVT). It is well known that several chronic or acute diseases or some clinical status, other than cirrhosis or hepatocellular carcinoma, are considered risk factors for abdominal thrombosis, as recently reviewed by Parikh et al. [3] who found, underlying the etiology of PVT, two orders of causes classified in local (including all known diseases associated with PVT) and systemic (including congenital thrombophilia) orders. For these reasons we planned this prospective study, in which patients with abdominal thrombosis, without liver cirrhosis or hepatocellular carcinoma, were included and compared with blood bank donors. The following four THRGFs PAI-1, MTHFR677, V Leiden 506Q, and Prothrombin 20210A were analyzed.

## 2. Material and Methods

### 2.1. Subjects

All Caucasian patients consecutively observed in our department with PVT and Budd-Chiari syndrome (BCS) from January 2005 to June 2011 were included. All patients with cirrhosis and/or hepatocellular carcinoma and patients with neoplasm other than myeloproliferative neoplasm (MPN) were excluded. As controls 150 Caucasian blood bank donors, consecutively observed in the same period were included. A total of 85 patients were consecutively enrolled, specifically 54 PVT and 31 BCS (10 patients with BCS and PVT were analyzed in the BCS group). To identify the presence of any disease recognized as a risk factor for abdominal thrombosis, a questionnaire in order to study underlying local risk factor according to Parikh et al. [3] was administered to the patients; any previous diagnosis of the following acute or chronic diseases or clinical status was registered: abdominal surgery or oral contraception or pregnancy or abdominal acute disease in the last three months, presence of MPN, or chronic disease recognized as a risk factor for abdominal thrombosis (Crohn's disease, Bechet's syndrome, Gaucher's syndrome, paroxysmal nocturnal hemoglobinuria, hemophagocytic syndrome, and nephrotic syndrome). Presence of thrombosis in other regions of the body and the extension of abdominal thrombosis (mesenteric, splenic or cava vein involvement) were also registered. All patients underwent gastroscopy and size of esophageal varices was recorded as large-medium/small/absent. All 235 subjects were asked about previous bleeding episodes.

This study protocol was approved by the local human research committee.

### 2.2. Abdominal Thrombosis: Diagnosis Criteria

PVT diagnosis was accepted when unambiguous diagnostic evidence for extrahepatic obstruction was detected by proper imaging techniques (Doppler ultrasound, computerized tomography, or magnetic resonance imaging).

BCS was diagnosed when unambiguous evidence for hepatic venous outflow obstruction at any point between the level of the small hepatic veins and the entrance of the inferior vena cava into the right atrium was detected by proper imaging techniques, as defined above. The presence of mesenteric vein thrombosis, spleen vein thrombosis, and cava thrombosis was also evaluated.

### 2.3. Thrombophilic Genetic Factors and Definition of Thrombophilia

To evaluate the role of these THRGFs in abdominal thrombosis, genotyping of polymorphisms of PAI-1, MTHFR677, V Leiden 506Q, and Prothrombin 20210A mutations was performed by PCR-RFLP according to Primignani and Mannucci [4], in heterozygous and homozygous status. We defined genetic thrombophilia as the presence of at least 1 of the following THRGFs PAI-1 4G-4G, MTHFR677TT, V Leiden Q506, and Prothrombin 20210A as in our previous studies [1, 2]. The diagnosis of MPN was performed according to 2001 World Health Organization Classification of Hematopoietic and Lymphoid Neoplasm [5].

All patients signed an informed consent and the study conformed to the ethical guidelines of the 1975 Helsinki Declaration.

### 2.4. Statistical Analysis

Any THRGF in patients with abdominal thrombosis was compared with that in healthy controls, using odds ratio with 95% confidence interval (OR 95% CI) and *χ* ^2^ test. Moreover the observed frequencies for THRGF genotypes were compared with those predicted in a population by the Hardy-Weinberg equilibrium, using a *χ* ^2^ test, both in patients with abdominal thrombosis and in healthy controls, using interactive web-tool system calculator [6]. OR 95% CI analysis and *χ* ^2^ test with *P* value were performed using Interactive Statistical Calculation Pages [7].

## 3. Results 

The whole group consisted of 235 subjects: 85 patients with abdominal thrombosis, of which 54 with PVT and 31 with BCS, and 150 blood bank donors.

Table 1 shows main demographic and clinical characteristics of patients with abdominal thrombosis (both PVT and BCS) and healthy controls. In the table also detailed clinical characteristics of 54 PVT and 31 BCS patients are reported; mesenteric and/or splenic vein thrombosis was present in 20 patients with abdominal thrombosis (15 with PVT and 5 BCS associated with PVT) and cava vein thrombosis in 7 BCS patients (3 associated with PVT). Large and medium esophageal varices were present in 47/85 patients, with a similar distribution in patients with PVT and BCS. A total of 23 patients have had one or more bleeding episodes, more frequently in PVT than in BCS, and 11 patients showed thrombosis in other sites.

A total of 75 patients with abdominal thrombosis showed associated disease or particular clinical status (46 PVT and 29 BCS): 37 MPN (20 PVT and 17 BCS), 12 abdominal surgery (10 PVT and 2 BCS), 10 oral contraception or pregnancy (6 PVT and 4 BCS), 7 abdominal acute disease (6 PVT and 1 BCS), and 9 chronic disease, specifically 3 Crohn's disease, 2 Bechet's syndrome, 1 Gaucher's syndrome, 1 paroxysmal nocturnal hemoglobinuria, 1 hemophagocytic syndrome, 1 nephrotic syndrome (4 PVT and 5 BCS). Ten patients (8 PVT and 2 BCS) and all 150 blood bank donors did not show any associated disease or particular clinical status, as defined above.

We found only one V Leiden 506Q homozygosis in a patient with PVT and not a single homozygosis for Prothrombin 20210A was found in the study. The prevalence of PAI-1 polymorphisms 4G-4G, 4G-5G, and 5G-5G was, respectively, 47 (55.3%), 22 (25.9%), and 16 (18.8%) in patients with abdominal thrombosis and 26 (17.3%), 64 (42.7%), and 60 (40.0%) in healthy controls.

The prevalence of MTHFR polymorphisms 677TT, C677T, and CC677 was, respectively, 24 (28.2%), 30 (35.3%), and 31 (36.5%) in patients with abdominal thrombosis and 20 (13.3%), 81 (54.0%), and 49 (32.7%) in healthy controls.

As a consequence of these polymorphism distribution is, PAI-1 4G-4G and MTHFR677TT deviated from that expected from a population in the Hardy-Weinberg equilibrium (PAI-1: *χ* ^2^ = 13.8, *P* < 0.001; MTHFR677: *χ* ^2^ = 7.1, *P* < 0.01) in patients with abdominal thrombosis, whereas the Hardy-Weinberg equilibrium was respected (*P* > 0.05 ) in healthy controls. V Leiden Q506 and Prothrombin 20210A were in the Hardy-Weinberg equilibrium (*P* > 0.05) both in patients with abdominal thrombosis and healthy controls.

In Table 2, OR (95% CI) of PAI-1 4G-4G, MTHFR677TT, V Leiden Q506, and Prothrombin 20210A, in relation to the presence of abdominal thrombosis, and detailed data in PVT and BCS patients are reported. In patients with abdominal thrombosis significant prevalence of PAI-1 4G-4G, MTHFR677TT, and V Leiden Q506 was found, and PAI-1 4G-4G was the most frequent when compared with healthy controls.

Table 3 shows detailed frequencies of THRGFs, PAI-1 4G-4G, MTHFRC677TT, V Leiden 506Q, and Prothrombin 20210A, in the 85 patients with abdominal thrombosis. A total of 28/37 (75.7%) patients with MPN showed presence of at least 1 THRGF, 10/12 (83.3%) with recent abdominal surgery, and 100% of the other 36 patients with oral contraception or pregnancy or chronic disease or abdominal acute disease or without a single disease or clinical status associated.

## 4. Discussion 

Our data represent, to our knowledge, the only study to date of these four THRGFs in abdominal thrombosis, evaluated in a consecutive series of patients in the same study.

The most relevant result of the present study was the pivotal role of PAI-1 4G-4G and MTHFR677TT in this series of Caucasian patients with abdominal thrombosis without liver cirrhosis or hepatocellular carcinoma; PAI-1 4G-4G has come out as the lead factor in the total abdominal thrombosis group, with similar prevalence in PVT (30/54) and BCS (17/31), confirming its important role in pathogenesis of portal thrombosis, as found by Balta et al. [8], and confirming itself as a thrombotic risk factor in different target organs, as showed by Tsantes et al. [9]. The control group study contains 26/150 (17.3%) PAI-1 4G-4G, and this data is very similar to a recent Italian study by Palmirotta et al. [10], showing 10/50 (20.0%) PAI-1 4G-4G prevalence in healthy subjects.

MTHFR677TT was significantly correlated with abdominal thrombosis mainly in PVT (16/54 patients). The study control group contained 20/150 (13.3%) MTHFR677TT homozygosis; other European studies on Caucasian population found MTHFR677TT prevalence from 10 to 13% in the control groups of three studies, focused on the association between this polymorphism and susceptibility to cervical cancer [11–13].

Both PAI-1 4G-4G and MTHFR677TT polymorphisms were not in the Hardy-Weinberg equilibrium in patients with abdominal thrombosis whereas the equilibrium was respected in the healthy controls. The imbalance of PAI-1 4G-4G and MTHFR677TT allele frequencies in patients with abdominal thrombosis, according to the Hardy-Weinberg law, is further evidence of possible pathogenetic role of these polymorphisms in abdominal thrombosis.

V Leiden 506Q was significantly correlated with abdominal thrombosis, with a particular prevalence in BCS patients (9/31 patients), confirming other previous articles on BCS [14, 15]; the Hardy-Weinberg equilibrium for V Leiden 506Q was respected (*P* > 0.05) both in patients with abdominal thrombosis and in healthy controls.

We failed to demonstrate a significant role of Prothrombin 20210A, in contrast to Primignani et al.'s study [16], who found this THRGF to be significantly correlated with abdominal thrombosis; in our study Prothrombin 20210A polymorphism was in the Hardy-Weinberg equilibrium both in patients with abdominal thrombosis and in healthy controls.

The multiple association of clinical and genetic factors, related to abdominal thrombosis, was the other very relevant point of our study. A total of 75 patients with abdominal thrombosis showed the association of some disease or particular clinical status investigated; 15/75 patients showed more than 1 THRGF. It is noteworthy that 6/10 patients without any associated disease or clinical status showed the association of more than one THRGF.

Analyzing specifically PVT patients, 37/54 patients showed the association of more causes of thrombosis: 13/20 with MPN and 23/26 patients with other associated disease or particular clinical status showed thrombophilia (1 patient more than one THRGF). All 8 patients without any associated disease or clinical status showed presence of thrombophilia and 5 of them more than one THRGF.

In BCS the presence of more causes of thrombosis was found: nearly all (30/31) patients showed this association: 16/17 with MPN and all 12 patients with other associated disease or particular clinical status showed thrombophilia (4 patients more than one THRGF). All 2 patients without any associated disease or clinical status showed presence of thrombophilia and 1 of them more than one THRGF.

These figures confirm those of the European Network for Vascular Disorders of the Liver [17] showing that 84% of BCS patients presented at least one, and many (46%) more than one, clinical and genetic thrombotic risk factor.

Analyzing specifically the 10 patients with PVT and BCS, MPN was present in 7 and chronic disease in 3; 9/10 showed at least one THRGF: 6 patients PAI-1 4G-4G, 2 patients MTHFR677TT, and 2 patients V Leiden 506Q; one patient showed the presence of more than one THRGF.

In our study PAI-1 4G-4G was the most frequent THRGF in patients with abdominal thrombosis, present in 18/37 patients with MPN, 23/38 patients with associated disease or particular clinical status, and 6/10 patients without any association. These values are much higher, compared to the presence of multiple risk factors in 15% of patients with splanchnic vein thrombosis as reported by Murad et al. [18].

The observed data confirmed the hypothesis that the development of thrombosis needs one or more agents as Bittencourt et al. [19] suggested: the convergence of vessel wall injury and/or venous stasis, known as local triggering factors, and the occurrence of acquired and/or inherited thrombophilia, also known as systemic risk factors.

THRGF screening, with the aim of the possible identification of patients who will develop thrombosis, could be useful in some acute or chronic diseases well known as risk factors for deep vein thrombosis or in pregnancy or during oral contraception therapy; in these patients anticoagulant therapy perhaps could be evaluated.

The present study indicates two main results: first, the pivotal role of PAI-1 4G-4G and MTHFR677TT in abdominal thrombosis and second the synergic role of more than one thrombophilic condition (both the association of clinical causes and genetic thrombophilia and the presence of more than one THRGF). The hypothesis that the genetic bases of thrombophilia could have a role in the pathogenesis of the diseases frequently associated with abdominal thrombosis, needs future confirmation.

In conclusion we remark that genetic thrombophilia, present in near 90% of patients with abdominal thrombosis in our study, and mainly PAI-1 4G-4G and MTHFR677TT play a significant role in thrombotic events in abdominal thrombosis with associated thrombophilic clinical conditions. We suppose that these allele polymorphisms may increase the inflammation response, which is the biomolecular base for both acute and chronic diseases, participating in endothelial dysfunction and abnormal angiogenesis, recently considered new perspectives for developing more effective treatment strategies in portal hypertension [20].

However, to assess this, larger longitudinal studies, hopefully from diverse geographical areas, are widely needed to confirm the interrelations of all pathogenic factors involved in the complex mechanism of abdominal thrombosis.

## Figures and Tables

**Table 1:** Main demographic and clinical characteristics of patients with abdominal thrombosis (AT) and healthy controls (HC). Portal vein thrombosis (PVT) and Budd-Chiari syndrome (BCS) patient characteristics are separately reported.

	(A) PVT (%)	(B) BCS (%)	(A) + (B) AT (%)	HC (%)
Number of patients	54 (100)	31 (100)	85 (100)	150 (100)
Age: median	53	46	51	53
Male sex	28 (51.9)	14 (45.0)	42 (49.4)	71 (47.3)
Mesenteric and/or splenic vein thrombosis	15 (27.8)	5 (16.1)	20 (23.5)	—
Cava vein thrombosis	0 (0)	7 (22.6)	7 (8.2)	0 (0)
Esophageal varices:	16/14/24	12/6/13	28/20/37	—
large-medium/small/absent	(29.6)/(25.9)/(44.5)	(38.7)/(19.4)/(41.9)	(32.9)/(23.6)/(43.5)	
Bleeding episodes	16 (29.6)	7 (22.6)	23 (27.0)	0 (0)
Thrombosis in other sites	5 (9.3)	6 (19.4)	11 (12.9)	0 (0)
Myeloproliferative neoplasm	20 (37.0)	17 (54.8)	37 (43.5)	0 (0)
Recent abdominal surgery	10 (18.5)	2 (6.4)	12 (14.1)	0 (0)
Oral contraception or pregnancy	6 (11.1)	4 (11.8)	10 (11.8)	0 (0)
Abdominal acute disease	6 (11.1)	1 (3.2)	7 (8.2)	0 (0)
Chronic disease	4 (7.4)	5 (16.1)	9 (10.6)	0 (0)
Not a single disease or clinical status associated	8 (14.8)	2 (6.4)	10 (11.8)	0 (0)

**Table 2:** Frequencies of thrombophilic genetic factors (THRGFs), PAI-1 4G-4G, MTHFR677TT, V Leiden 506Q, and Prothrombin 20210A, in patients with abdominal thrombosis (AT) and healthy controls (HC). Frequencies in patients with portal vein thrombosis (PVT) and Budd-Chiari syndrome (BCS) are separately reported.

	PVT (%)	BCS (%)	(A) AT (%)	(B) HC (%)	OR (95% CI) (A) versus (B)	*χ* ^2^ test: *P* value (A) versus (B)
Number of patients	54 (100)	31 (100)	85 (100)	150 (100)	—	—
PAI-1 4G-4G	30 (55.5)	17 (54.8)	47 (55.3)	26 (17.3)	6.8 (3.5–13.2)	41.3: *P* < 0.000
MTHFR677TT	16 (28.6)	8 (25.8)	24 (28.2)	20 (13.3)	2.6 (1.3–5.3)	7.9: *P* = 0.005
V Leiden 506Q	4 (7.4)	9 (29.0)	13 (15.3)	7 (4.6)	3.7 (1.3–10.8)	7.9: *P* = 0.005
Prothrombin 20210A	4 (7.4)	1 (3.2)	5 (5.8)	4 (2.6)	2.3 (0.5–2.4)	1.5: *P* = 0.2
>1 THRGF	10 (18.5)	5 (16.2)	15 (17.6)	2 (1.3)	15.9 (3.3–103.4)	21.5: *P* < 0.000
At least 1 THRGF	44 (81.5)	30 (96.7)	73 (87.1)	54 (36.0)	10.8 (5.2–23.1)	54.4: *P* < 0.000

**Table 3:** Thrombophilic genetic factors (THRGFs), PAI-1 4G-4G, MTHFR677TT, V Leiden 506Q, and Prothrombin 20210A, in patients with abdominal thrombosis with myeloproliferative neoplasm (MPN), recent abdominal surgery (RAS), oral contraception or pregnancy (OCP), chronic disease (CD), abdominal acute disease (AAD), and it a single disease or clinical status associated (NODCSA).

	MPN (%)	RAS (%)	OCP (%)	AAD (%)	CD (%)	NODCSA (%)	Total
Number of patients	37 (100)	12 (100)	10 (100)	7 (100)	9 (100)	10 (100)	85 (100)
PAI-1 4G-4G	18 (48.6)	7 (58.3)	6 (60)	4 (57.1)	6 (66.6)	6 (60.0)	47 (55.3)
MTHFR677TT	8 (21.6)	2 (16.6)	3 (30)	2 (28.5)	3 (33.3)	6 (60.0)	24 (28.2)
V Leiden 506Q	6 (16.2)	1 (8.3)	3 (30)	0 (0)	0 (0)	3 (30.0)	13 (15.3)
Prothrombin 20210A	2 (5.4)	0 (0)	0 (0)	1 (14.3)	0 (0)	2 (20.0)	5 (5.8)
>1 THRGF	5 (13.5)	1 (8.3)	2 (20.0)	0 (0)	1 (11.1)	6 (60.0)	15 (17.6)
At least 1 THRGF	28 (75.7)	10 (83.3)	10 (100)	7 (100)	9 (100)	10 (100)	73 (87.1)
